# Prognostic value of microRNA-21 in epithelial ovarian carcinoma: A protocol for systematic review and meta analysis

**DOI:** 10.1097/MD.0000000000023849

**Published:** 2020-12-24

**Authors:** Kun Ji, Xiaohua Wang, Anqi Zhang, Hongwei Wen

**Affiliations:** a Department of Clinical Laboratory, Liaocheng people's Hospital; b Department of Clinical Laboratory, Liaocheng Fourth People's Hospital; c Department of Central Laboratory; d Department of Reproductive Endocrinology Laboratory, Reproductive Medicine, Liaocheng people's Hospital, Liaocheng, Shandong Province, P.R. China.

**Keywords:** epithelial ovarian carcinoma, meta-analysis, miRNA-21, prognosis

## Abstract

**Backgroud:**

The expression of microRNA-21 has been shown to be associated with the prognosis in patients with malignant tumors. However, its prognostic value in epithelial ovarian carcinoma (EOC) remains controversial. This meta-analysis aimed to synthesize available data to clarify the association between microRNA-21 expression levels and clinical prognosis in EOC patients.

**Methods:**

Eligible literatures were searched from Embase, Google Scholar, PubMed, Web of Science, Medline, Cochrane Library, China Scientific Journal Database, China National Knowledge Infrastructure, Chinese BioMedical Database and Wanfang Database to identify eligible studies. Papers in English or Chinese published from their inception to November 2020 will be included. Methodological quality for each eligible trial will be assessed by using the Newcastle-Ottawa Quality Assessment Scale. Odds ratios or hazards ratios with corresponding 95% confidence intervals were pooled to estimate the prognosis value of microRNA-21 by using Stata 14.0 and Review Manager 5.3 software.

**Results:**

This study will provide a high-quality evidence-based medical evidence of the correlations between microRNA-21 expression and overall survival and disease-free survival.

**Conclusion:**

The findings of this systematic review will show the effect of high expression of microRNA-21 on the prognosis of EOC patients.

**Trial registration number:**

INPLASY2020110064

## 1 Introduction

Ovarian carcinoma (OC) is the 19th most commonly diagnosed malignancy and the second most common cause of gynecologic cancer death in women (accounts for about 20% of all female reproductive cancers) around the world.^[1–4]^ According to global cancer statistics, about 184,800 deaths occurred worldwide in 2018, ranking the 15th leading cause of tumor-related deaths.^[1,2]^ OC can occur at any age, more common in patients older than 50 years.^[3–6]^ With increasing life-expectancy, the number of cases diagnosed each year is increasing.^[3–6]^ Epithelial ovarian carcinoma (EOC) is the primary type of OC, accounting for more than 90% of total OC.^[7–11]^ Despite the improvement of diagnostic and therapeutic methods in the past decades, the prognosis of EOC remains unsatisfactory.^[5,6,10,11]^ Over 60% to 70% of OC patients are diagnosed at advanced stage, with 5-year survival rates below 45%.^[9]^ Therefore, the development of a novel biological marker for the prognosis prediction of EOC remains urgent.

MicroRNA-21, a member of the microRNA family, is encoded by the MIR21 gene located on chromosome 17q23.2 in humans.^[12,13]^ The mature microRNA-21 is formed from endogenous non-coding RNA molecules with a length of ∼22 nucleotides.^[13]^ It can bind with the 3’UTR sequence of messenger RNA (mRNA) to degrade mRNA or inhibit the transcription of mRNA, thereby participating in the biological processes of regulating cell proliferation, apoptosis and innate immunity.^[13–15]^ It may be involved as an oncogene or tumor suppressor gene in the occurrence and development of various tumors including EOC.^[13–18]^ Previous studies reported that microRNA-21 is unregulated in EOC and can regulate the growth, metastasis and apoptosis of cancer cells through altering the expression of various target molecules, such as programmed cell death 4, phosphate and tension homolog (PTEN), reversion-inducing-cysteine -rich protein with Kazal motifs (RECK) gene, and B-cell lymphoma-2 (Bcl-2).^[7,19–23]^ Although several studies have investigated the potential value of microRNA-21 expression in the prognosis prediction and diagnosis of EOC, the exact association between microRNA-21 and survival in patients with EOC has not yet been systematically evaluated.^[17,18]^ In order to solve the issue, our study will used meta-analysis to evaluate the effect of high expression of microRNA-21 on the prognosis of EOC patients.

### 1.1 Review question

Whether the high expression of microRNA-21 is in association with poor prognosis in patients with EOC?

### 1.2 Study aim/Objective

This study will try to explore the effect of high expression of microRNA-21 on the prognosis of EOC patients.

## 2 Methods

### 2.1 Study registration

This meta-analysis protocol is based on the Preferred Reporting Items for Systematic Reviews and meta-analysis Protocols (PRISMA-P) statement guidelines.^[24]^ The PRISMA-P checklist for the protocol is provided in the PRISMAP-checklist. The protocol of the systematic review has been registered on the International Platform of Registered Systematic Review and Meta-Analysis Protocols (INPLASY). The registration number was INPLASY2020110064 (URL: https://inplasy.com/inplasy-2020-11-0064/). This meta-analysis is a secondary research which based on some previously published data. Therefore, the ethical approval or informed consent was not required in this study.

### 2.2 Search strategy

The retrieval strategy will be created based on discussion of all the researchers on the basis of the Cochrane handbook guidelines. The plan searched terms are as follows: “ovarian carcinoma” or “epithelial ovarian carcinoma” or “oophoroma” or “OC” or “EOC” and “microRNA-21” or “miR-21” and “prognostic” or “survival”. The detailed sample of search strategy for PubMed database is shown in Table 1. Similar search strategies will be modified and used for the other databases.

**Table 1:** Searching strategy in PubMed.

Search Strategy
#1. “microRNA-21” or “miRNA-21” or “miR-21” [Title/Abstract].
#2. “Ovarian cancer” or “Ovarian tumor” or “Ovarian neoplasm” or “Ovarian carcinoma” or “Ovarian malignant” or “Ovarian oncology” or “Epithelial ovarian cancer” or “Epithelial ovarian tumor” or “Epithelial ovarian neoplasm” or “Epithelial ovarian carcinoma” or “Epithelial ovarian malignant” or “Epithelial ovarian oncology” or “oophoroma” or “Cancer of the ovarian” or “Cancer of the epithelial ovarian” or “OC” or “EOC” [Title/Abstract].
#3. “Ovarian cancer” [MeSH].
#4. #2 or #3.
#5. “Survival” [Title/Abstract]
#6. “Prognosis” [Title/Abstract]
#7. #5 or #6
#8. #1 and #4 and #7
#9. Limit #8 to human
#10. Limit #9 to yr=”-November 2020”

### 2.3 Information sources

Electronic databases including Embase, Google Scholar, PubMed, Web of Science, Medline, Cochrane Library, China Scientific Journal Database, China National Knowledge Infrastructure, Chinese BioMedical Database and Wanfang Database, will be systematically searched for eligible literatures from their inception to November 2020. Language is limited with English and Chinese.

### 2.4 Eligibility criteria

#### 2.4.1 Types of studies

All available controlled trials that assessed the effect of high expression of microRNA-21 on overall survival (OS) and disease-free survival (DFS) of patients diagnosed with EOC will be included in this systematic review; The included studies should provide the relationship between miRNA-21 expression and clinical pathological characteristics.

#### 2.4.2 Types of participants

Patients must be diagnosed with EOC based on pathology and histology. No restrictions regarding age, gender, racial, region, education and economic status in this analysis. Patients with other malignancies are not included.

#### 2.4.3 Types of interventions

In the experimental group, serum microRNA-21 expression levels were detected in all EOC patients confirmed by histopathology.

#### 2.4.4 Comparator

In the control group, the expression levels of serum microRNA-21 were detected in normal participants.

#### 2.4.5 Exclusion criteria

Articles without sufficient available data, animal experiments, case reports and series, literature reviews, meta-analysis, letters, conference abstract, and other unrelated studies will be all excluded from analysis.

### 2.5 Types of outcome measures

(1) Overall survival (OS, which is defined as the time from the date of randomization to death from any cause);
(2) DFS, which is the time from date of random assignment to date of recurrence or death);
(3) Hazard ratios with corresponding 95% confidence intervals will be extracted from trials or be estimated from Kaplan-Meier survival curves by established methods.^[25]^

### 2.6 Study selection and data extraction

#### 2.6.1 Study selection

**Figure 1:**
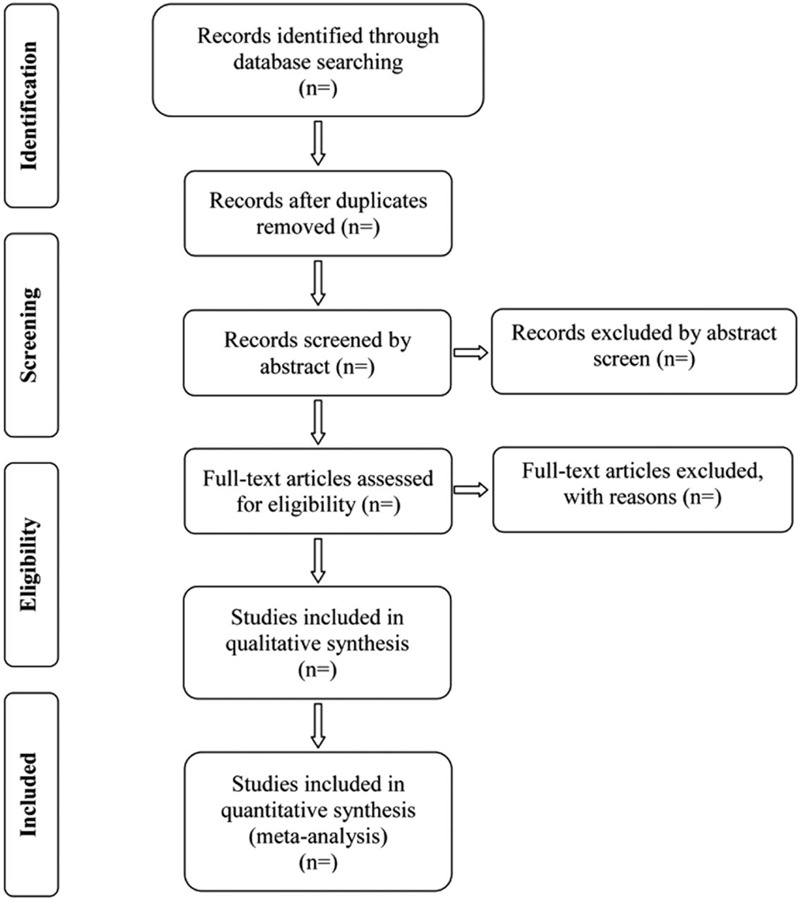
Study selection process for the meta-analysis.

Endnote X7 software will be used for literature managing and records searching. Two experienced authors (Kun Ji and Xiaohua Wang) will be reviewed independently to identify potential trials by assessing the titles and abstracts. The full text will be further reviewed to determine potential eligible studies. A PRISMA-compliant flow chart (Fig. 1) will be used to describe the selection process of eligible trials. Excluded studies and reasons for exclusion will be recorded. Disagreements between the 2 researchers will be resolved by consensus or by a third independent investigator (Anqi Zhang).

#### 2.6.2 Data extraction

Two investigators (Kun Ji and Xiaohua Wang) will be responsible for the data extraction independently. Information extracted from eligible literatures is shown in Table 2. When any data are missing or insufficient, we will contact original authors by using email. If the data is not available, we will only analyze the currently available data and discuss its potential impact.

**Table 2:** Information extracted from eligible literatures.

Classification	Parameters
Study characteristics	First author name, year of publication, country of study, sample size, microRNA-21 detection method, et al.
Participant characteristics	Age, gender, race, tumor stage, inclusion and exclusion criteria, et al.
Outcome and other data	Overall survival (OS), Disease-free survival (DFS), hazard ratios (HRs) with corresponding 95% confidence intervals (CIs), et al.

### 2.7 Risk of bias assessment

Two experienced authors (Kun Ji and Xiaohua Wang) will assess the risk of bias for each eligible study by using the Newcastle-Ottawa Quality Assessment Scale independently.^[26]^ Newcastle-Ottawa Quality Assessment Scale comprise 3 quality parameters including selection, comparability, and result evaluation. Each study was scored from 0 to 9 according to these parameters, and ≥7 were judged to be of higher quality. Any disagreements will be resolved via discussion with a third researcher (Anqi Zhang).

### 2.8 Statistical analysis

Stata 14.0 (Stata Corp., College Station, TX, USA) and Review Manager 5.3 (Nordic Cochran Centre, Copenhagen, Denmark) statistical software were used for statistical analyses. Cochran's Q and Higgins *I*^*2*^ statistic were used to assess heterogeneity among the included clinical trials. *P* < .1 for the Chi^2^ statistic or an *I*^*2*^ > 50% will be considered as showing considerable heterogeneity.^[27]^ A fixed effect model will be used to calculate the outcomes when statistical heterogeneity is absent; otherwise, the random effects model will be used for analysis. Odds ratio or hazard ratios with corresponding 95% confidence intervals was used to evaluate the relationship between microRNA-21 expression and OS and DFS.

### 2.9 Subgroup analysis

Subgroup analysis will only be performed if sufficient clinical data is available. It will be conducted to explore the source of heterogeneity based on different race, EOC stages, microRNA-21 detection method, and survival data source.

### 2.10 Sensitivity analysis

Sensitivity analysis of each parameter was carried out by 1-by-1 elimination method to assess the reliability and robustness of the aggregation results. A summary table will report the results of the sensitivity analyses.

### 2.11 Additional analysis

#### 2.11.1 Publication bias analysis

If the included studies are sufficient (≥10 trials), we will detect publication biases of included trials using funnel plots, Begg's and Egger regression test.^[28–30]^ If publication bias existed, a trim-and-fill method should be applied to adjust the pooled odds ratio.^[31]^

#### 2.11.2 Evidence evaluation

The guidelines of the Grading of Recommendations, Assessment, Development, and Evaluation will be used to assess the quality of evidence and the strength of the main result recommendations.^[32]^

### 2.12 Dissemination

We will disseminate the results of this systematic review by publishing the manuscript in a peer-reviewed journal or presenting the findings at a relevant conference.

## 3 Discussion

Although the diagnosis and treatment methods for EOC has been greatly developed in recent decades, its 5-year survival rate still not been significantly improved.^[9–11]^ Therefore, finding biomarkers with high specificity and high sensitivity has important clinical significance for the prognosis of EOC.^[33]^ MicroRNA-21 is overexpressed in many malignant tumors and has been implicated in tumorigenesis.^[34,35]^ Accumulating evidence supports a central role for the microRNA-21 in EOC initiation, progression, and chemoresistance.^[16–18,34,35]^ Báez-Vega et al^[34]^ found that the expression of microRNA-21 in EOC serum is remarkably higher than that in non-EOC serum. Chan et al^[18]^ Pointed out that microRNA-21 is closely related to the occurrence, drug resistance and prognosis of tumors, and found that women with tumors that overexpressed microRNA-21 were associated with a shorter progression-free survival. MicroRNA-21 can also regulate drug resistance via apoptosis and cellular survival pathways. Recently, Xu's research ^[17]^ showed that the increased serum microRNA-21 expression was correlated with advanced EOC stage, high tumor grade, and shortened OS. All these findings indicate that serum microRNA-21 may serve as a novel prognostic marker, and can be used as a therapeutic target for the treatment of EOC. All in all, we hope that this meta-analysis will provide more accurate and objective evidence for the relationship between microRNA-21 expression and prognosis in EOC patients.

The systematic review will also have some limitations. Language bias may exist due to the limitation of English and Chinese studies. In addition, the detection method of microRNA-21 and patient age, tumor stage may be different among the included trials. Therefore, there may be a risk of heterogeneity.

## Author contributions

**Conceptualization:** Hongwei Wen and Kun Ji

**Data curation:** Kun Ji and Xiaohua Wang

**Formal analysis:** Kun Ji, Xiaohua Wang and Anqi Zhang

**Funding acquisition:** Anqi Zhang

**Investigation:** Kun Ji, Xiaohua Wang and Anqi Zhang

**Methodology:** Kun Ji, Xiaohua Wang and Anqi Zhang

**Project administration:** Hongwei Wen

**Resources:** Hongwei Wen and Kun Ji

**Software:** Hongwei Wen and Kun Ji

**Supervision:** Hongwei Wen and Kun Ji

**Validation:** Hongwei Wen and Anqi Zhang

**Visualization:** Kun Ji and Xiaohua Wang

**Writing – original draft:** Kun Ji and Xiaohua Wang

**Writing – review & editing**: Hongwei Wen and Anqi Zhang
